# Cavity-mediated iSWAP oscillations between distant spins

**DOI:** 10.1038/s41567-024-02694-8

**Published:** 2024-12-09

**Authors:** Jurgen Dijkema, Xiao Xue, Patrick Harvey-Collard, Maximilian Rimbach-Russ, Sander L. de Snoo, Guoji Zheng, Amir Sammak, Giordano Scappucci, Lieven M. K. Vandersypen

**Affiliations:** 1 QuTech and Kavli Institute of Nanoscience, Delft University of Technology, Delft, Netherlands; 2 QuTech and Netherlands Organization for Applied Scientific Research (TNO), Delft, Netherlands

**Keywords:** Qubits, Single photons and quantum effects, Quantum information, Superconducting devices, Quantum dots

## Abstract

Direct interactions between quantum particles naturally fall off with distance. However, future quantum computing architectures are likely to require interaction mechanisms between qubits across a range of length scales. In this work, we demonstrate a coherent interaction between two semiconductor spin qubits 250 μm apart using a superconducting resonator. This separation is several orders of magnitude larger than for the commonly used direct interaction mechanisms in this platform. We operate the system in a regime in which the resonator mediates a spin–spin coupling through virtual photons. We report the anti-phase oscillations of the populations of the two spins with controllable frequency. The observations are consistent with iSWAP oscillations of the spin qubits, and suggest that entangling operations are possible in 10 ns. These results hold promise for scalable networks of spin qubit modules on a chip.

## Abstract (web summary)

Coupling semiconductor spin qubits over long distances using a superconducting resonator makes different quantum architectures possible. Now, the coherent swapping of quantum states has been observed between qubits coupled using this design.

## Main

Solving relevant problems with quantum computers will require millions of error-corrected qubits^1^. Efforts across quantum computing platforms based on superconducting qubits, trapped ions and colour centres target a modular architecture for overcoming the obstacles to scaling, with modules on separate chips or boards, or even in separate vacuum chambers or refrigerators^2^. For semiconductor spin qubits^3,4^, the small qubit footprint together with the capabilities of advanced semiconductor manufacturing^5,6^ may enable a large-scale modular processor integrated on a single chip^7^.

Semiconductor spin qubits are most commonly realized by confining individual electrons or holes in electrostatically defined quantum dots. Nearly all quantum logic demonstrations between such qubits are based on the exchange interaction that arises from wavefunction overlap between charges in neighbouring dots, with typical qubit separations of 100–200 nm (refs. ^4,8^). The monolithic integration of a million-qubit register at a 100 nm pitch will face challenges, related to the fan-out of control and readout wires. Combining local exchange-based gates and operations between qubits 10 μm to 250 μm apart provides a path to on-chip interconnected modules^7,9^. Various approaches for two-qubit operations over larger distances have been pursued, such as coupling spins via an intermediate quantum dot^10,11^, capacitive coupling^12^ and shuttling of electrons, propelled either by gate voltages^13,14^ or by surface acoustic waves^15^. However, the first two methods are still limited to submicrometre distances and the use of surface acoustic waves faces many practical obstacles, especially in group IV semiconductors. Electrically controlled shuttling, although regarded as a promising route, is relatively slow, and therefore, the coupling distance is constrained by the relevant coherence times. On the other hand, integrating spin qubits with on-chip superconducting resonators using the circuit quantum electrodynamics framework provides an elegant way of constructing an on-chip network^16,17^. With this approach, coupling distances of several hundreds of micrometres are achievable and operations can be just as fast as those based on wavefunction overlap.

In recent years, strong spin–photon coupling^18–20^ and resonant spin–photon–spin coupling^21^ have both been reported in hybrid dot–resonator devices. Among the many ways for constructing distant quantum gates in this architecture^17^, coupling the spins dispersively via virtual photons looks highly promising^22,23^. In this regime, the frequencies of the two qubits are detuned from the resonator frequency and the leakage of quantum information into resonator photons is largely suppressed^24,25^. Spin–spin coupling in the dispersive regime has been observed recently in spectroscopy^26^, but two-qubit oscillations in the time domain remains to be demonstrated. Furthermore, in colour centres^27^ and self-assembled quantum dots^28^, the observation of cavity-mediated spin–spin evolution presents an outstanding challenge.

In this work, we demonstrate the time-domain control of a dot–resonator–dot system and realize two-qubit iSWAP oscillations between distant spin qubits. The two qubits are encoded in single-electron spin states and they are coupled via a 250-μm-long superconducting NbTiN on-chip resonator. The resonator is also used for dispersively probing the spin states^18,29^. First, we demonstrate operations on individual spin qubits at the flopping-mode operating point^30,31^ and characterize the corresponding coherence times. Then, we realize iSWAP oscillations between the two distant spin qubits in the dispersive regime. We study how the oscillation frequency varies with spin–cavity detuning, spin–photon coupling strength and frequency detuning between the two spin qubits, and compare the results with theoretical simulations.

## Device

**Fig. 1:**
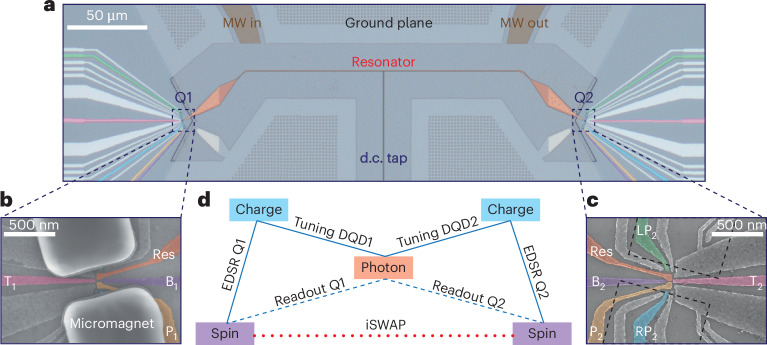
Dot–resonator–dot device. **a**, False-coloured optical image of the device that is used in this work, showing the resonator, ground planes and gate fan-out of the DQDs. The microwave (MW) in/out ports are used to probe the transmission through the resonator, and the d.c. tap is used to bias the shared top-gate Res (in **b** and **c**) of the two DQDs. **b**, Scanning electron microscopy image of a DQD with the same design as the measured DQD_1_, showing the gate pattern and micromagnets on top. **c**, Scanning electron microscopy image of a similar DQD without micromagnets to show the full gate layout of DQD_2_. The dashed lines outline the micromagnets of the measured device. The false-coloured gates in **b** and **c** are plunger gates Res and P_*i*_, tunnel-barrier gates B_*i*_ and T_*i*_, and side gates RP_*i*_ and LP_*i*_. **d**, Schematic of the distant spin–spin coupling architecture. The blue and red lines indicate the dispersive and resonant couplings, respectively. The solid lines represent direct couplings, which are the charge–photon electric-dipole coupling and the spin–charge coupling enabled by the micromagnet gradients. These couplings are utilized for probing the DQD charge susceptibility, which allows us to tune the DQD to the degeneracy point and for electric-dipole spin resonance, respectively. The dashed lines indicate the indirect spin–photon couplings that enable dispersive spin readout. The dotted line indicates a second-order indirect coupling, that is, the spin–spin coupling mediated by virtual photons, which enables iSWAP oscillations.

The device is fabricated on a ^28^Si/SiGe heterostructure and was used in a previous experiment to demonstrate dispersive spin–spin coupling via spectroscopic measurements^26^. It contains an on-chip superconducting resonator with an impedance of 3 kΩ (ref. ^32^) and a double quantum dot (DQD) at both ends, with gate filters^33^ (Fig. 1a). The resonator is etched out of a 5- to 7-nm thick NbTiN film, and is 250 μm long. Its fundamental half-wave mode, with *ω*_r_/2π = 6.9105 GHz and a linewidth of *κ*_r_/2π = 1.8 MHz (*Q* ≈ 3,800), is used in the experiment for both long-range coupling and dispersive spin readout. The DQD is defined by a single layer of Al gate electrodes (Fig. 1c). The gates labelled Res are galvanically connected to the resonator. On top of each DQD, a pair of cobalt micromagnets is deposited (Fig. 1b). The device is mounted on a printed circuit board attached to the mixing chamber of a dilution refrigerator with a base temperature of 8 mK (Supplementary Section B provides additional details on the experimental setup).

We accumulate electrons in the DQD_*i*_ (*i* = 1, 2) via plunger gates Res and P_*i*_. The interdot tunnel couplings are controlled by the tunnel-barrier gates, labelled T_*i*_ and B_*i*_. The side gates RP_*i*_ and LP_*i*_ are used to control the electrochemical potentials and thus the detuning for each DQD. In the time-domain experiments, we pulse the detunings via the RP_*i*_ gates and drive single qubits by applying microwave bursts through the LP_*i*_ gates. The d.c. voltages on these gates are chosen such that DQD_1_ is at the degeneracy point between the (1,0)–(0,1) charge configuration, whereas DQD_2_ is at (3,2)–(2,3) to reach the desired tunnel coupling with modest gate voltages ((*m*, *n*) indicate the number of electrons in each DQD). Both DQDs are tuned to a similar tunnel coupling of ~4.8 GHz. At the charge degeneracy point, the electron is delocalized between the two dots and the resulting charge dipole is maximized^34^, enabling a charge–photon coupling strength of ~192 MHz for both DQDs. The magnetic-field gradient produced by the micromagnets results in the hybridization of spin and charge states, which allows an indirect electric-dipole interaction between the photons and spins (Fig. 1d). The spin–photon coupling can be effectively switched off by pulsing the detuning via the RP_*i*_ gate, to an operating point at which the electron is tightly localized in a single dot and its electric susceptibility is suppressed. The micromagnets are tilted by ±15° relative to the double-dot axis, which permits tuning the Zeeman energy difference between the two spin qubits by rotating the external magnetic field^21^. In the presence of an external magnetic field *B*_ext_ of 50 mT and at an angle of 4.7°, both qubits are set to a frequency of ~6.82 GHz, slightly detuned from the resonator frequency. The two spins then resonantly interact with each other, mediated by virtual photons in the resonator (Fig. 1d).

## Flopping-mode qubits

We first separately examine the individual qubits located at both ends of the resonator. We encode the qubits in two eigenstates at the charge degeneracy point: |–, ↓〉 as logical |0〉 and *α*|–, ↑〉 + *β*|+, ↓〉 as logical |1〉. Here |↓〉/|↑〉 is the spin state, |–〉/|+〉 is the bonding/antibonding orbital and the coefficient *α*/*β* ≫ 1 is determined by the degree of spin–charge hybridization^35,36^ ($\left\vert \pm \right\rangle =\left(\left\vert L\right\rangle \pm \left\vert R\right\rangle \right)/\sqrt{2}$, where |*L*〉/|*R*〉 indicates the state in which an electron occupies the left/right dot, respectively). The qubits are manipulated using electric-dipole spin resonance (EDSR) enabled by the micromagnets^37^. Operation at the charge degeneracy point, chosen to maximize the charge–photon and spin–photon coupling strengths, implies that even the EDSR Rabi frequencies are maximized, since they rely on the same matrix element (Fig. 1d). This regime is also known as the flopping-mode regime^31^. Furthermore, since the detuning between the spin and cavity is larger than the spin–photon coupling strength (as shown below), the spin qubit state is only weakly hybridized with the photons.

Spin readout is natively achieved in the regime of dispersive spin–photon coupling given that the resonator frequency depends on the electron’s spin state. To detect the spin states, we send a microwave probe signal to the resonator at a frequency corresponding to the resonator’s frequency with the qubit in |0〉. In all the measurements, the change in microwave transmission (termed transmission for short) relative to this reference value is used as a measure of the |1〉 population. Due to the limited qubit relaxation times (discussed later), the |1〉 signal decays within hundreds of nanoseconds. Even though we use a travelling-wave parametric amplifier^38^ to increase the signal-to-noise ratio, signal averaging over many cycles is needed (Supplementary Section A provides a detailed explanation of the read-out procedure).

For qubit initialization, we rely on spontaneous relaxation to the ground state. For this purpose, the short relaxation timescales at the charge degeneracy point are helpful. In practice, a waiting time of 1 μs is sufficient for initialization (≥5*T*_1_).

**Fig. 2:**
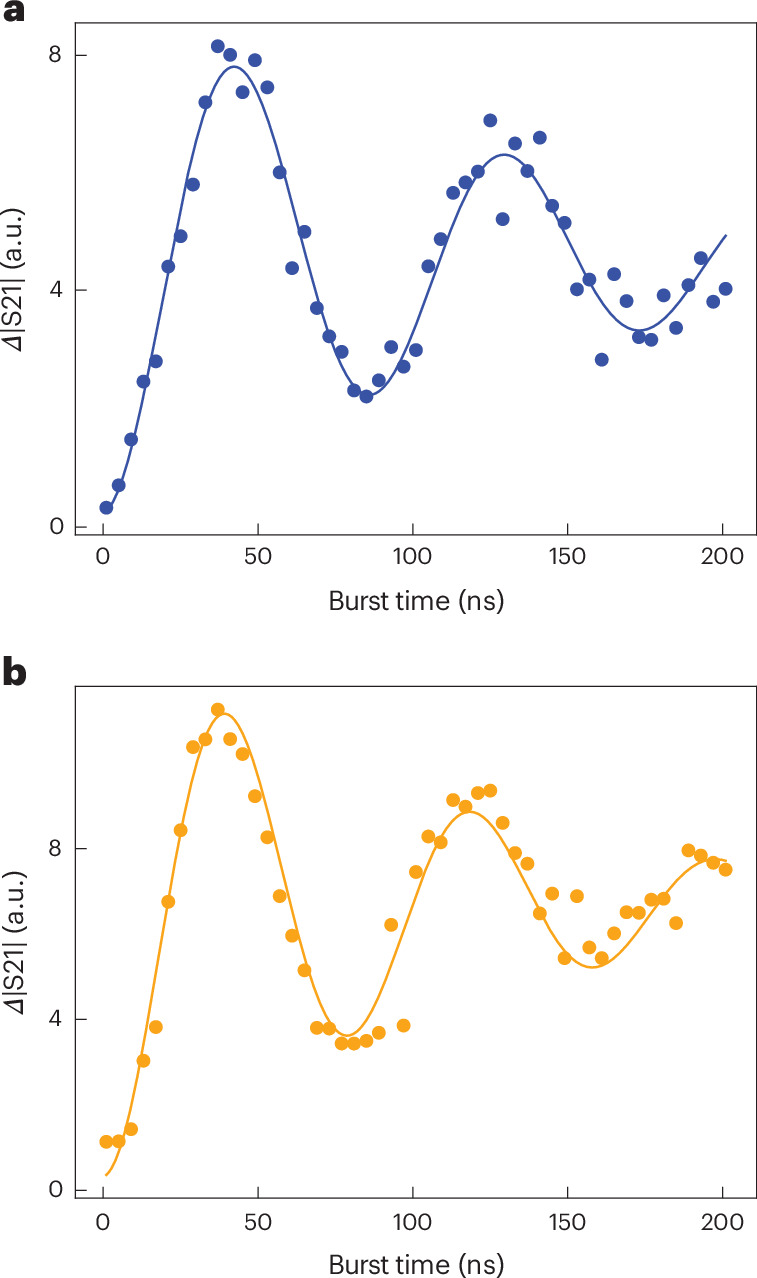
Rabi oscillations in flopping mode. **a**,**b**, Rabi oscillations for qubit 1 (**a**) and qubit 2 (**b**), driven in the flopping-mode regime. The measured transmission through the resonator as a function of the applied MW burst time, resonant with qubit 1 and qubit 2, is shown by the data points. The solid lines represent fits to the data with a damped sinusoid. From the fits, we extract a Rabi decay time ${T}_{2}^{{{\rm{Rabi}}}}$ of 100 ns and 120 ns for the two qubits. Although experimenting on one qubit, the other qubit is parked in the left dot of the corresponding DQD, and therefore, its coupling to the resonator is effectively switched off. Each data point is averaged for 10^5^ times. Source data

Time-domain single-qubit control of both qubits is illustrated in Fig. 2. We repeatedly initialize the qubit to |0〉, apply a resonant microwave burst of variable duration through gate LP_*i*_ and successively measure both qubits. Although we measure one qubit, we pulse the detuning of the other qubit away from the charge degeneracy point so that it does not affect the transmission through the resonator. When we plot the average transmission versus microwave burst time, we observe a damped oscillation, as expected.

**Extended Data Fig. 1:**
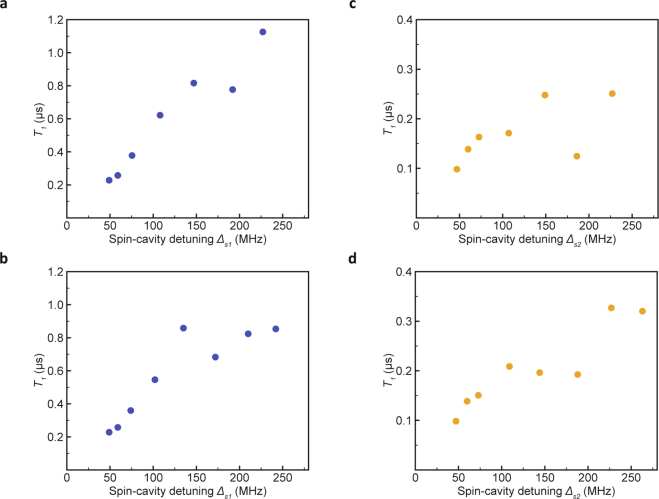
Relaxation time *T*_1_ as a function of spin-cavity detuning *Δ*_*s*_ with the cavity loaded with one charge only. **a,c**. Measured *T*_1_ of qubit 1(**a**) and 2(**c**) as function of *Δ*_*s*_ while recalibrating the magnetic field angle *β*_*ϕ*_ for every *Δ*_*s*_ value in order to maintain the two spin frequencies approximately equal for all *T*_1_ measurements. **b,d**. Measured *T*_1_ of qubit 1(**b**) and 2(**d**) as function of *Δ*_*s*_ with the calibrated angle of *β*_*ϕ*_ = 10^∘^ to equalize the spin frequencies at a *Δ*_*s*,1(2)_ of 50 MHz and leaving it unchanged for all other *T*_1_ measurements. Since the Purcell decay rate is proportional to ${g}_{s,1(2)}^{2}/{\Delta }_{s,1(2)}^{2}$, its effect should be similar for both qubits since we have *g*_*s*,1_ ≈ *g*_*s*,2_ and *Δ*_*s*,1_ ≈ *Δ*_*s*,2_. We observe that the measured *T*_1_ time indeed degrades for smaller spin-cavity detuning, which is a sign of Purcell-decay. However, even though the qubits share the same spin-cavity detuning and spin-photon coupling, the measured *T*_1_ times of the two qubits differ by up to a factor of 4. Therefore, we suspect Purcell decay to have an influence on the relaxation time but do not expect it to be the only source of *T*_1_ decay. Note that these data are collected in a separate cool-down from the numbers measured in Table S1, which explains the difference between them. Source data

Using standard pulse sequences, we next characterize the relaxation and decoherence times of the spin qubits in this system (Supplementary Table 1). The relaxation (*T*_1_) and dephasing (${T}_{2}^{\,*}$) times, which range from 100 ns to 260 ns and 40 ns to 80 ns, respectively, do not match the state-of-the-art spin qubit benchmarks^39^. This is expected given the strong spin–charge hybridization at the charge degeneracy point, making the qubits highly sensitive to charge noise^30^. This is the flip side of the faster Rabi oscillations and stronger spin–photon coupling at this working point. Additional contributions to relaxation arise from the Purcell decay induced by the resonator^40^ (Extended Data Fig. 1), and additional decoherence sources include the residual photon population induced by the readout signals^41^ and intrinsic slow electrical drift in this device.

## Two-qubit interaction

The spin–spin interaction in the dispersive regime, with both spins resonant with each other but detuned from the resonator, is described by the Tavis–Cummings Hamiltonian^42^. This Hamiltonian describes the collective qubit coupling with a resonator and can be simplified to the following dispersive spin–spin coupling Hamiltonian in the rotating-wave approximation^22,43^:

1 $$H\approx \hslash J\left({\sigma }_{+,1}{\sigma }_{-,2}+{\sigma }_{-,1}{\sigma }_{+,2}\right),$$

where *J* is the effective spin–spin coupling strength and *σ*_±,*i*_ denote the usual raising and lowering operators for qubit *i*. The coupling strength is given by

2 $$2J={g}_{{\rm{s}},1}{g}_{{\rm{s}},2}\left(\frac{1}{{\varDelta }_{2{\rm{s}},1}}+\frac{1}{{\varDelta }_{2{\rm{s}},2}}\right),$$

where *g*_s,1_ and *g*_s,2_ are the spin–photon coupling strengths for spins 1 and 2, respectively. *Δ*_2s,1(2)_ describes the detuning between the frequency of qubit 1 (2) and the loaded cavity frequency. The interaction with the two charge dipoles dispersively shifts the cavity frequency away from its bare frequency, to ~6.884 GHz when both electrons interact with the cavity (both DQDs at charge degeneracy) and to ~6.897 GHz when only one electron is coupled^26^.

**Fig. 3:**
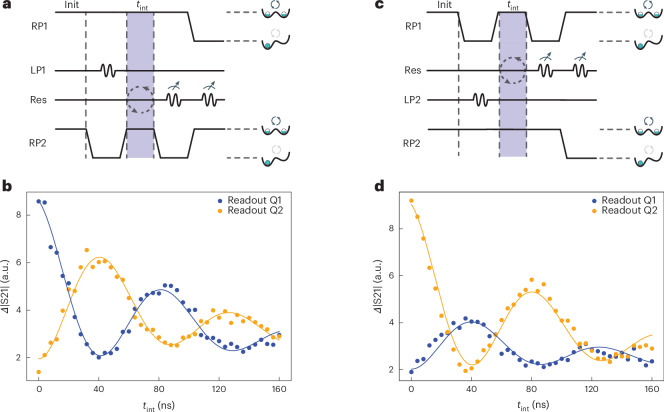
iSWAP oscillations between two distant spin qubits. **a**, Pulse sequence for the measurement of iSWAP oscillations starting from |10〉. The two qubits are initialized in |00〉 by relaxation at the charge degeneracy point. Then, qubit 2 is isolated in the left dot for 500 ns, during which a π-pulse is applied to qubit 1. After 10 ns, qubit 2 is pulsed back into interaction, and both spins interact with each other for *t*_int_. After the interaction interval, qubit 2 is isolated in its left dot for 1 μs, where relaxation is expected to be slower, whereas qubit 1 is read out using a 400-ns-long probe tone. Subsequently, qubit 1 is isolated in its left dot and qubit 2 is read out using a 400 ns probe tone. **b**, The data points show the measured (change in) transmission, representative of the spin-up population for each spin, starting from |10〉. Each data point is averaged for 10^6^ times. Here *g*_s,1(2)_/2π ≈ 21.5 MHz and *Δ*_2s,1(2)_/2π ≈ 65.5 MHz. The solid lines represent fits to the dispersive Hamiltonian model of equation (1) (with noise added; Supplementary Section D), from which we extract an interaction strength 2*J*/2π of 11.6 ± 0.2 MHz. **c**, Pulse sequence similar to that in **a**, but starting from |01〉. **d**, Similar data and fit as in **b**, but starting from |01〉. Here we extract 2*J*/2π = 11.8 ± 0.2 MHz. Source data

**Extended Data Fig. 2:**
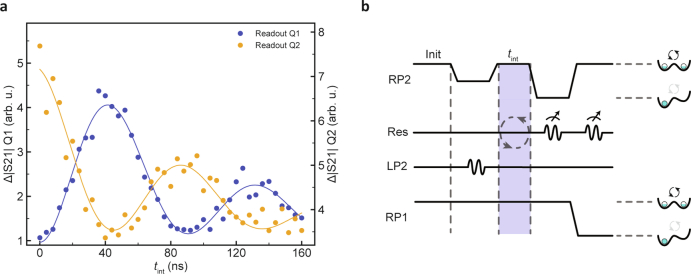
SWAP oscillations between the two distant spins, using single-dot EDSR instead of the flopping-mode regime to excite qubit 2. **a**. The operation regime is similar as in Fig. 3b,d, with *g*_*s*,1(2)_/2*π* ≈ 21.5 MHz and *Δ*_2*s*,1(2)_/2*π* ≈ 65.5 MHz. The solid lines represent fits to the dispersive Hamiltonian model of Eq. (1), from which we extract an interaction strength 2*J*/2*π* of 11.1 MHz. Note the visibility of qubit 2 is off-set compared to all other measurements (both traces being plotted on different scales). The exact mechanism is unknown, but we have verified that it is caused by the increased power of the microwave burst necessary to drive the qubit in a single dot. Each data point is averaged for 10^6^ times. **b**. Pulse sequence similar to Fig. 3a, with the difference being that the MW drive is now resonant with qubit 2 when pulsed to a single dot, to prepare $\left\vert 01\right\rangle$. The data points in **a** are averaged for 10^6^ times. Source data

The Hamiltonian of equation (1) generates iSWAP oscillations between the two spins. We probe this dynamics operating for now in a regime in which *g*_s,1(2)_/2π ≈ 21.5 MHz and *Δ*_2s,1(2)_/2π ≈ 65.5 MHz. Both spins are initialized to |0〉 by waiting for 1 μs at zero detuning (Fig. 3a,c). Next, one of the spins is prepared in |1〉, using a calibrated π-pulse in the flopping mode, whereas the other spin is pulsed away from charge degeneracy to effectively decouple it from the cavity (Extended Data Fig. 2 shows the initialization in a single dot). The spins are then allowed to interact with each other by pulsing the second spin back to charge degeneracy, at which point the spins are resonant with each other but still detuned from the (loaded) cavity. After a variable interaction time *t*_int_, the spins are read out sequentially, once again with the other spin decoupled from the cavity.

**Extended Data Fig. 3:**
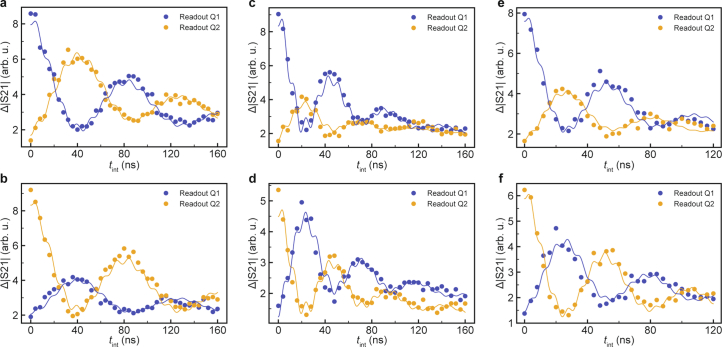
Two-qubit interaction data fitted with the full model. The rapid oscillations arising from the fits are due to coherent energy exchange with the resonator via residual vacuum Rabi oscillations. The fitted spin-photon coupling strength *g*_*s*_/2*π*, the spin-spin interaction strength 2*J*/2*π* and the estimated fidelities for the $\sqrt{{{\rm{iSWAP}}}}$ gate ${F}_{\sqrt{{{\rm{iSWAP}}}}}$ and the iSWAP gate FiSWAP are given hereby. **a**. ${g}_{s}/2\pi =20.9\,{{\rm{MHz}}},2J/2\pi =11.5\,{{\rm{MHz}}},{F}_{\sqrt{{{\rm{iSWAP}}}}}=82.2 \% ,{F}_{{{\rm{iSWAP}}}}=67.4 \%$. **b**. ${g}_{s}/2\pi =21.0\,{{\rm{MHz}}},2J/2\pi =11.6\,{{\rm{MHz}}},{F}_{\sqrt{{{\rm{iSWAP}}}}}=84.0 \% ,{F}_{{{\rm{iSWAP}}}}=72.2 \%$. **c**. ${g}_{s}/2\pi =29.7\,{{\rm{MHz}}},2J/2\pi =21.2\,{{\rm{MHz}}},{F}_{\sqrt{{{\rm{iSWAP}}}}}=76.5 \% ,{F}_{{{\rm{iSWAP}}}}=62.0 \%$. **d**. ${g}_{s}/2\pi =29.4\,{{\rm{MHz}}},2J/2\pi =20.9\,{{\rm{MHz}}},{F}_{\sqrt{{{\rm{iSWAP}}}}}=77.5 \% ,{F}_{{{\rm{iSWAP}}}}=63.2 \%$**e**. ${g}_{s}/2\pi =31.0\,{{\rm{MHz}}},2J/2\pi =18.0\,{{\rm{MHz}}},{F}_{\sqrt{{{\rm{iSWAP}}}}}=80.6 \% ,{F}_{{{\rm{iSWAP}}}}=66.2 \%$**f**. ${g}_{s}/2\pi =31.6\,{{\rm{MHz}}},2J/2\pi =18.7\,{{\rm{MHz}}},{F}_{\sqrt{{{\rm{iSWAP}}}}}=82.5 \% ,{F}_{{{\rm{iSWAP}}}}=70.3 \%$. The estimated $\sqrt{{{\rm{iSWAP}}}}$ gate fidelity reported in the main text is an average over the the numbers from **a** and **b**. Source data

Figure 3b(d) shows the measured evolution of both spins starting from |10〉 (|01〉). The populations evolve periodically in anti-phase in both experiments, as expected for coherent iSWAP oscillations. The extracted oscillation frequencies are ~11.7 MHz (Fig. 3b,d). The populations of the two spins, separated by more than 200 μm, are exchanged in just ~42 ns. A coupling time of ~21 ns is expected to maximally entangle the spins, based on equation (1). The fidelity of the entangling operation in this regime is numerically estimated to be 83.1% (Extended Data Fig. 3 and Supplementary Section D). The number of visible periods is limited due to the comparatively fast decoherence. Also, because *T*_1_ is only a factor of 2 to 3 longer than *T*_2_, the oscillations are damped asymmetrically towards the ground state. The unequal visibilities of the readout for the two qubits can be attributed to two causes. First, the dominating reason is that the qubit relaxation times differ, which impacts the signal accumulated during the 400 ns probe interval. Second, the dispersive shift in the resonator frequency has a different magnitude (*Δ*∣S21∣) for the two qubits.

**Fig. 4:**
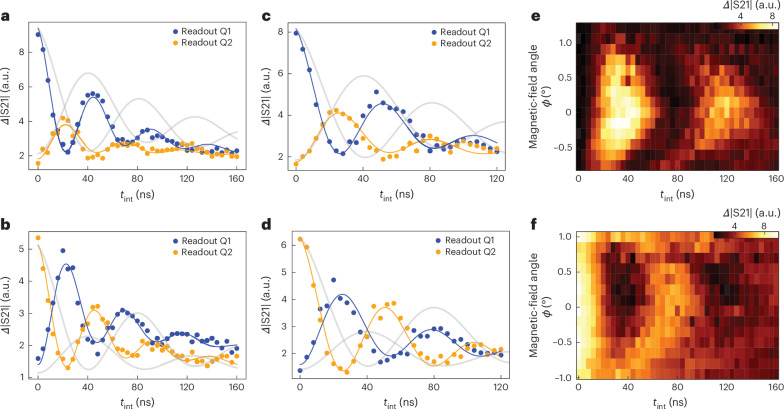
Control of iSWAP oscillations. **a**,**b**, iSWAP oscillations similar to that shown in Fig. 3b,d, with *g*_s,1(2)_/2π increased to ~31.9 MHz. The fitted oscillation frequencies are now 21.4 ± 0.3 MHz (**a**) and 21.3 ± 0.3 MHz (**b**). **c**,**d**, iSWAP oscillations similar to that shown in Fig. 3b,d, with *g*_s,1(2)_/2π increased to ~31.9 MHz and *Δ*_2s,1(2)_/2π increased to ~89 MHz. Here the fitted frequencies are 18.2 ± 0.4 MHz (**c**) and 18.7 ± 0.3 MHz (**d**). In **a**–**d**, the data points and solid lines represent the measurements and the fit, as shown in Fig. 3. The faint solid lines reproduce the (rescaled) solid lines from Fig. 3, for comparison. The data points in **a**–**d** are averaged for 10^6^ times. **e**, iSWAP oscillations as a function of the magnetic-field angle *ϕ* with *B*_r_ = 52.3 mT, starting from |01〉 and reading out qubit 1. A chevron pattern is visible, as discussed in the main text. **f**, iSWAP oscillations as a function of the magnetic-field angle similar to that in **e** but reading out qubit 2. The *ϕ* axes for **e** and **f** are set to *ϕ* = 0 at which the oscillation is the slowest. The actual *ϕ* = 0 points do not match, possibly due to a slight difference in the micromagnet magnetization between the measurements for **e** and **f**. The data points in **e** and **f** are averaged for 4.5 × 10^5^ times. Source data

**Extended Data Fig. 4:**
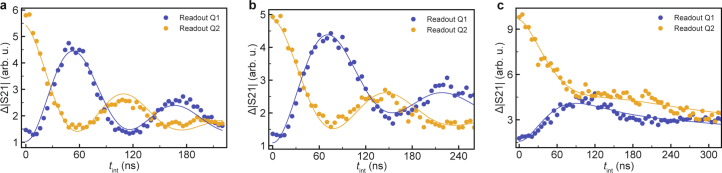
iSWAP oscillations between the two distant spins for increasing *β* = *Δ*_2*s*_/*g*_*d*_. **a**–**c**. The spin-photon coupling *g*_*s*,1(2)_/2*π* is similar as in Fig. 3 for all panels (~ 21.5 MHz), but *Δ*_*s*,1(2)_/2*π* is changed to 110 MHz (*β* ~ 5), 150 MHz (*β* ~ 7) and 220 MHz (*β* ~ 10) for **a**–**c**, respectively. The solid lines represent fits to the dispersive Hamiltonian model of Eq. (1), from which we extract the respective interaction strengths 2*J*/2*π* of 8.7 MHz, 6.7 MHz and 5.6 MHz. For *β* = 10, not only the oscillation is slower compared the case of *β* = 3, but the spin dephasing time was reduced to 20–25 ns in this case, for reasons that are unclear (perhaps a charge fluctuator was activated here). This results in a significantly damped oscillation which makes it difficult to perform a proper fit as one can observe in panel **c**. The data points in **a,c** are averaged for $3\cdot 1{0}^{6}$ times and in **b** for 2 ⋅ 10^6^ times. Source data

Next, we test whether the measured oscillation frequency varies with the control parameters according to equation (2). First, we increase the spin–photon coupling strength *g*_s,1(2)_/2π from ~21.5 MHz to ~31.9 MHz by reducing the tunnel couplings to ~4.35 GHz, keeping *B*_ext_ fixed to maintain approximately the same spin–cavity detuning as that shown in Fig. 3. The smaller tunnel coupling decreases the charge–photon detuning, thereby increasing the charge-induced dispersive shift and lowering the resonator frequency. Simultaneously, it reduces the spin frequency, due to increased spin–charge admixing. Therefore, the spin–cavity detuning stays almost the same as before, namely, *Δ*_2s,1(2)_/2π ≈ 63 MHz. As expected, we find an increased oscillation frequency of ~21.4 MHz (Fig. 4a,b). Next, we increase the spin–cavity detuning to *Δ*_2s,1(2)_/2π ≈ 89 MHz by reducing the external magnetic field *B*_ext_ from 50 mT to 49 mT, and keeping the spin–photon coupling strength the same as that shown in Fig. 4a,b. The angle of the field is re-calibrated to 11.5° to ensure that the two qubits are still on resonance with each other. In this setting, we find that the oscillation frequency is reduced from ~21.4 MHz to ~18.5 MHz (Fig. 4c,d). These fitted oscillation frequencies are slightly different than those predicted using equation (2). We expect that the discrepancy will decrease for a larger *Δ*_2s_/*g*_s_ ratio, that is, deeply in the dispersive regime (Extended Data Fig. 4 shows the data for *Δ*_2s_/*g*_s_ ≈ 5, 7 and 10). Consistent with this interpretation, the fitted frequencies are in excellent agreement with the values predicted by a more complete simulation, which takes into account the non-zero photon population in the resonator (Extended Data Fig. 3 and Supplementary Section D).

One further control knob is given by the frequency detuning between the two qubits, which can be adjusted via the external-magnetic-field angle *B*_*ϕ*_. The oscillation frequency is expected to vary according to $\frac{1}{2\uppi }\sqrt{{\left(2J\right)}^{2}+{\left({\omega }_{{\rm{Q}}1}-{\omega }_{{\rm{Q}}2}\right)}^{2}}$, where *ω*_Q*i*_ are the angular frequencies of the qubits (the spectroscopy data are given elsewhere^26^). As shown in Fig. 4e,f, the measurement results display chevron patterns as a function of the magnetic-field angle and the duration of the two-qubit interaction. For *ω*_Q1_ = *ω*_Q2_, the oscillation is the slowest and the |01〉 and |10〉 populations are maximally interchanged (maximum contrast). When the magnetic-field angle is changed such that *ω*_Q1_ differs from *ω*_Q2_, the rotation axis is tilted in the |01〉/|10〉 subspace and we observe accelerated oscillations but with lower contrast, as expected.

**Fig. 5:**
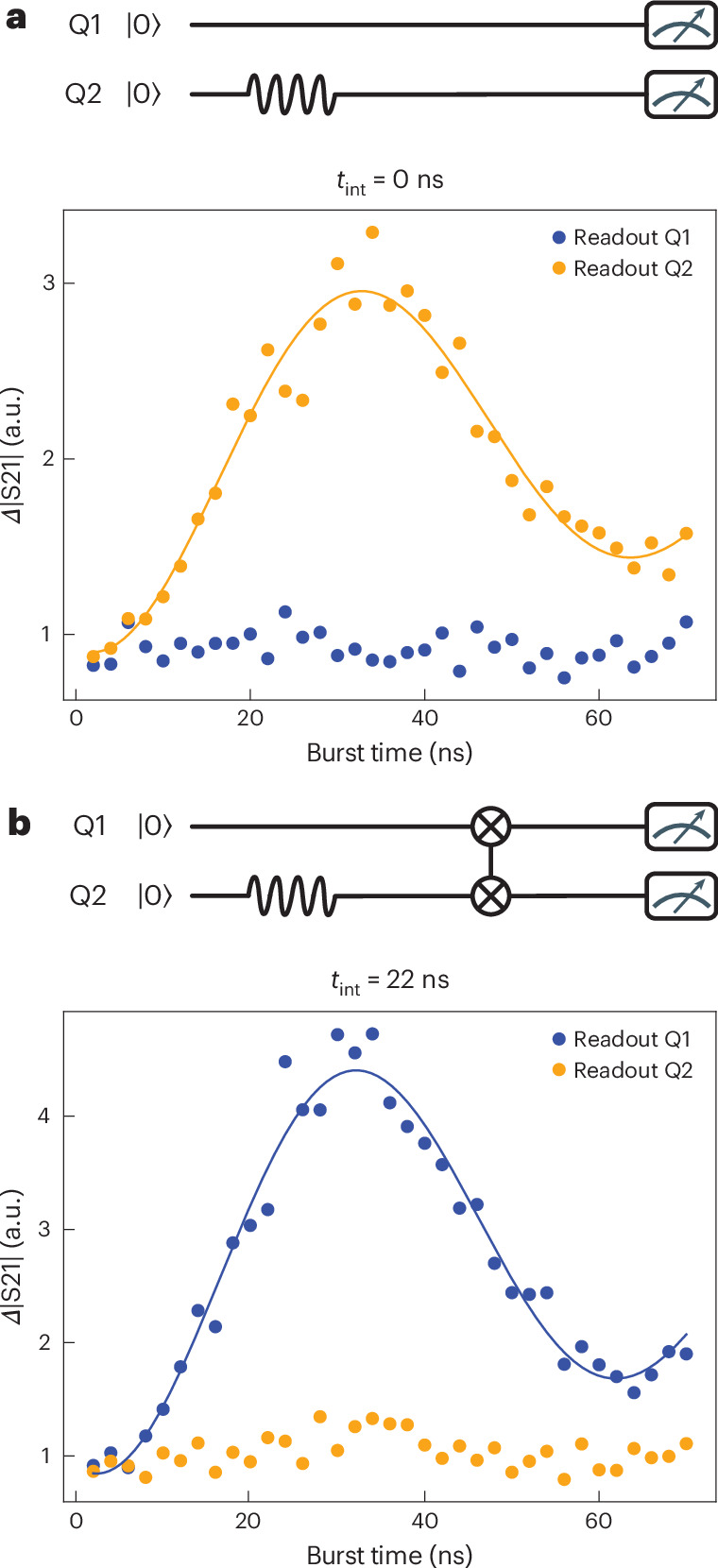
Swapped Rabi-cycle experiment. **a**, Rabi oscillation of qubit 2 serving as a reference experiment. Starting with both spins in the ground state, a microwave burst of variable duration is applied to qubit 2 followed by the read out of this qubit (next, the experiment is repeated and qubit 1 is read). The data points and solid lines represent the measurement and a fit to a sinusoid, as shown in Fig. 2. Each data point is averaged for 5 × 10^5^ times. **b**, Rabi oscillation of qubit 2 detected through qubit 1. After the microwave burst is applied to qubit 2, a calibrated iSWAP evolution is executed before read out (performed as that in **a**). Source data

Finally, we apply a calibrated iSWAP oscillation in a practical scenario in which the coherent time evolution of one qubit is transferred to the state of the other qubit. First, a reference experiment is executed (Fig. 5a), where we apply a resonant microwave burst of variable duration to qubit 2 in the flopping-mode regime and read out this qubit (orange data points), similar to that in Fig. 2b. Then, we repeat the same measurement but instead read out qubit 1 (Fig. 5a, blue data points). As shown in Fig. 5, qubit 2 completes a Rabi cycle and qubit 1 remains in the ground state. Next, we perform a similar experiment with a calibrated iSWAP evolution inserted after the microwave burst, which is expected to map the Rabi oscillation of qubit 2 onto qubit 1 by swapping their populations. As shown in Fig. 5b, the coherence expressed by the Rabi oscillation of qubit 2 is now indeed visible in the final state of qubit 1 (blue data points); meanwhile, qubit 2 arrives in the ground state, which is the initial state of qubit 1 (orange data points).

## Conclusion

Looking ahead, we aim to increase the quality factor of the oscillations in several ways. First, the charge qubit linewidth of ~60 MHz is much larger than that in state-of-the-art devices, where linewidths down to 2.6 MHz have been reported^44^. Given the admixing of the charge and spin degrees of freedom needed for spin–photon coupling, a narrower charge qubit linewidth immediately translates to a narrower spin qubit linewidth. Furthermore, the ~30 MHz spin–photon coupling strength can be enhanced to at least ~300 MHz via both stronger lever arms, higher resonator impedance and stronger intrinsic or engineered spin–orbit coupling^45^. Combining these will allow application in the deep dispersive regime without compromising on the gate speed. With this and a modest improvement in the resonator linewidth of 0.3 MHz, a >99% two-qubit gate fidelity should be within reach^22^.

Furthermore, high-fidelity single-qubit operation can be achieved by conventional electric-dipole spin resonance with the electron in a single dot^46^. Spin readout can be improved by including a third (auxiliary) dot for spin-to-charge conversion based on Pauli-spin blockade, enabling rapid and high-fidelity single-shot readout through the resonator^47^. With a dedicated readout resonator or a sensing dot, readout and two-qubit gates can be individually optimized. Scaling up the present two-qubit interaction requires local spin-frequency control and tunability, allowing the qubit frequencies to be individually tuned. Alternatively, the present architecture also allows for exploring two-qubit gates based on cross-resonance coupling or in the longitudinal-coupling regime.^48–51^

These results mark an important milestone in the effort towards the creation of on-chip networks of spin qubit registers. The increased interaction distance between qubits allows for the co-integration of classical electronics and for overcoming the wiring bottleneck. The networked qubit connectivity calls for the design of optimized quantum error correction codes. Moreover, this platform opens up new possibilities in quantum simulation involving both fermionic and bosonic degrees of freedom.

## Online content

Any methods, additional references, Nature Portfolio reporting summaries, source data, extended data, supplementary information, acknowledgements, peer review information; details of author contributions and competing interests; and statements of data and code availability are available at 10.1038/s41567-024-02694-8.

## Supplementary information

Supplementary Information Supplementary Sections A–D, Figs. 1–4 and Table 1.

Supplementary Data 1 Numerical source data for Supplementary Fig. 3.

Supplementary Data 2 Numerical source data for Supplementary Fig. 4.

## Source data

Source Data Fig. 2 Numerical source data for Fig. 2.

Source Data Fig. 3 Numerical source data for Fig. 3.

Source Data Fig. 4 Numerical source data for Fig. 4.

Source Data Fig. 5 Numerical source data for Fig. 5.

Source Data Extended Data Fig. 1 Numerical source data for Extended Data Fig. 1.

Source Data Extended Data Fig. 2 Numerical source data for Extended Data Fig. 2.

Source Data Extended Data Fig. 3 Numerical source data for Extended Data Fig. 3.

Source Data Extended Data Fig. 4 Numerical source data for Extended Data Fig. 4.

## Data Availability

Data supporting this work are available via 4TU.ResearchData at 10.4121/6c34cc01-bbab-4f7c-b2e8-6fab5f07020d.v3 (ref. ^52^). Source data are provided with this paper.

## Code availability

Codes used for data processing and numerical simulations are available via 4TU.ResearchData at 10.4121/6c34cc01-bbab-4f7c-b2e8-6fab5f07020d.v3 (ref. ^52^).
