# Supplementary material for: Cavity-mediated iSWAP oscillations between distant spins
Source: Nat Phys. 2024 Dec 9;21(1):168–74. doi: 10.1038/s41567-024-02694-8 (PMC11746143; doi:10.1038/s41567-024-02694-8)
Supplement: Supplementary file 1 — Supplementary Sections A–D, Figs. 1–4 and Table 1. [file 41567_2024_2694_MOESM1_ESM.pdf]

nature physics

Supplementary information https://doi.org/10.1038/s41567-024-02694-8

Cavity-mediated iSWAP oscillations between distant spins

In the format provided by the authors and unedited

A: Calibration and Readout Procedure

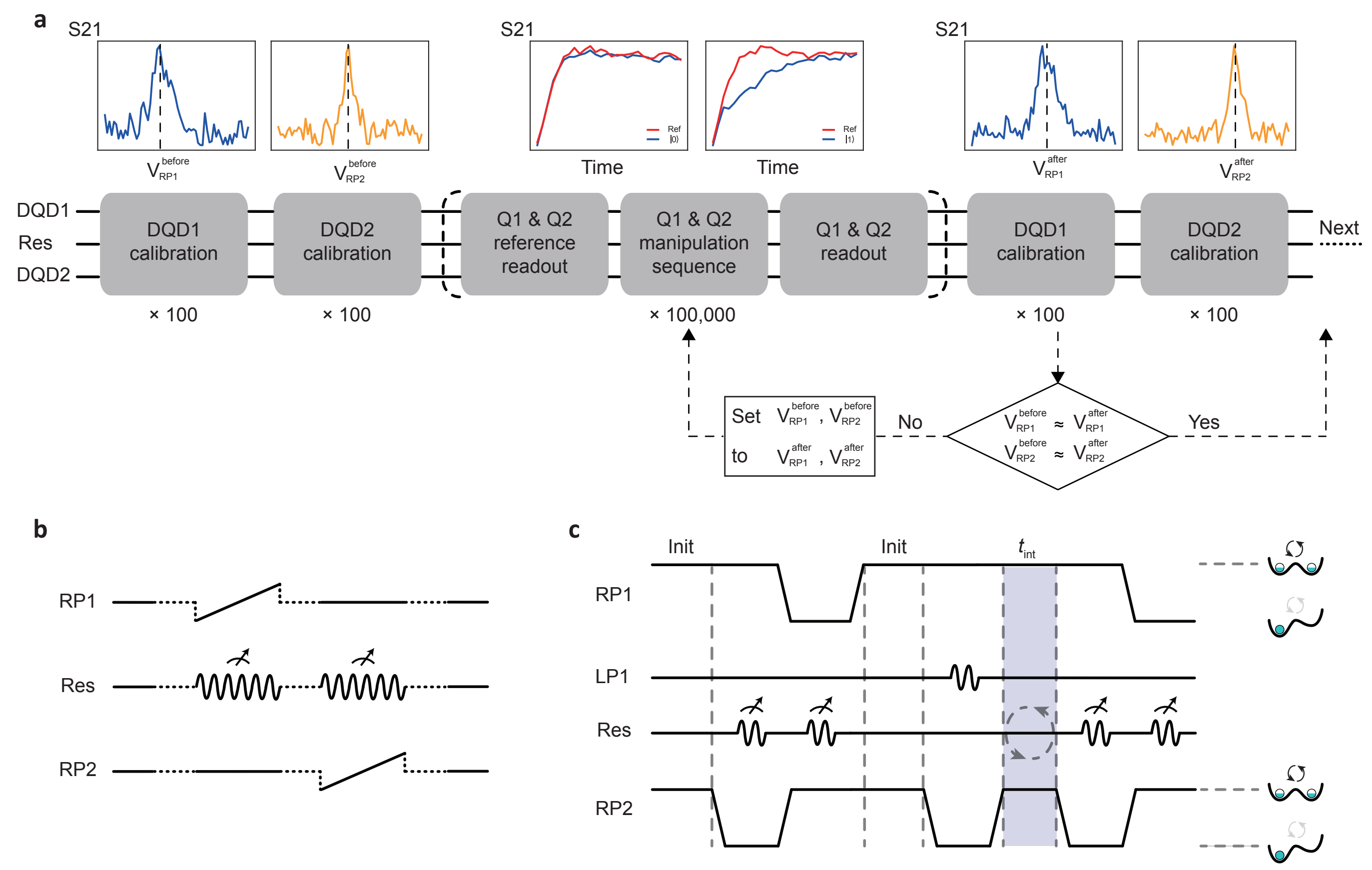

Fig. S1: Procedure for the calibration and measurement. **a.** Workflow of the two-qubit interaction measurement. The calibrations for the charge degeneracy points are executed before and after each measurement. The insets on top show example calibration results to find the correct RP_i voltages, where the charge susceptibility is largest. Each calibration scan is averaged for 100 times before moving to the next step. Each single-shot execution of the two-qubit experiment contains reference readout segments followed by a sequence of operations and readout segments for both qubits after the operations. This single-shot experiment is executed 100,000 times before the next round of calibrations. If the calibrations before and after suggest that no drift or only mild drift took place, the result of the 100,000 single-shot is accepted. Otherwise the data is retaken. Next, a control parameter is changed, for instance t_{int}, and another round of 100,000 repetitions is executed. After experiments have been completed for all values of the control parameter, the entire procedure is repeated. The final result is analyzed based on 10 such repetitions, thus on 1,000,000 averages. The averaged readout traces are divided into segments of 32 ns, each of which is processed with a fast Fourier transform. The signal persists for a few 100 ns, so we add up the FFT of 13 consecutive segments. The result is shown in the top-middle inset. The integrated difference between the reference readout trace and the readout trace after the sequence of operations on the qubits is recorded as $\Delta|S21|$ in the plots in the main text. **b.** Pulses used for the calibration of the RP_i voltages of the two qubits. The RP_i gate voltage is swept linearly for 4 μs in a range of ± 11 mV, while the resonator is being probed with a continuous wave of equal duration. The probe frequency is chosen to correspond to the resonance when the DQD is coupled to the resonator so the transmission signal is reduced when the electron is parked in a single dot and is at is maximum value when it is at the charge degeneracy point. **c.** Complete pulse sequence for measurements of two-qubit iSWAP oscillations, which is plotted in a simplified version in Fig. 3. Every ramp in used in all the pulse sequences are 5 ns long. Before the operations for qubit manipulation, each qubit is measured in its ground state to record the reference readout traces. The integration time is equal to that of the readout traces after the qubit manipulation.

Calibration

As described in Ref. [1], the device undergoes slow electric drift in experiment, which is reflected by the instability of the RP_i and LP_i voltages at the inter-dot transition point. To mitigate the effect of the drift, we implement automated calibration of the RP_1 and RP_2 voltages for the (1,0)-(0,1) and (3,2)-(2,3) transitions for DQD_1 and DQD_2 respectively. Note that the voltages applied to LP_1 and LP_2 are not calibrated, as any drift can be compensated by resetting the RP_1 and RP_2 voltages.

The sequence of operations for the two-qubit experiments is illustrated in Fig. S1a. We first apply continuous wave to probe the resonator transmission while linearly sweeping the RP_1 gate to calibrate the voltage required for the charge degeneracy point for DQD_1, followed by a similar calibration for DQD_2 (Fig. S1b). After these calibrations, the RP_1 and RP_2 voltages are set to the new values and recorded as $V_{\mathrm{RP}_1}^{\mathrm{before}}$ and $V_{\mathrm{RP}_2}^{\mathrm{before}}$. Then we apply pulses to control the two-qubit experiment, which includes a reference measurement to extract the readout signal for the $|0\rangle$ state of both qubits and the actual measurement after qubit manipulation (Fig. S1c). The integrated difference between the two readout traces is the measurement result and is shown as $\Delta|S21|$ in the plots in the main text. Here, the reference traces help to additionally compensate for drift. After the two-qubit experiment, the RP_1 and RP_2 voltages for the charge degeneracy points, $V_{\mathrm{RP}_1}^{\mathrm{after}}$ and $V_{\mathrm{RP}_2}^{\mathrm{after}}$, are calibrated and compared to $V_{\mathrm{RP}_1}^{\mathrm{before}}$ and $V_{\mathrm{RP}_2}^{\mathrm{before}}$. If their differences are both smaller than 0.3 mV, we proceed to the sequence for the next setting. Otherwise, the result is abandoned and the same measurement is retaken. It is important to note that the RP_i gates are located to the side of the dots and thus they have much smaller lever-arms compared to the top plunger gates. In a similar device, the lever-arm of a RP_i gate is measured to be 30 μeV/mV for the right dot and its lever-arm for the left dot is measured to be 17 μeV/mV. Thus, the lever-arm for the inter-dot detuning is only 13 μeV/mV.

For single-qubit experiments, the workflow is similar except that the calibration and reference readout are executed only for the qubit under investigation while the other qubit is parked in the left dot.

Readout

In the regime of dispersive spin-photon coupling, the frequency of the resonator depends on the state of the qubit. Therefore, the qubit state can be detected by probing the transmission of a readout tone through the resonator. The readout tone is generated at the frequency of the resonator when the qubit is in the $|0\rangle$ state. Thus, a high transmission signal amplitude corresponds to the $|0\rangle$ state whereas a low transmission signal amplitude corresponds to the $|1\rangle$ state. The two states can also be distinguished by the phase difference in the signals but we choose to use the amplitude for readout for its higher sensitivity in our operating regime.

Because of the magnetic field and the coupling to the spin, the linewidth of the resonator increases to $\sim$5 MHz , corresponding to a response time of $\sim$200 ns, comparable to the T_1's of the qubits. Given the limited signal-to-noise ratio obtained when averaging for a few 100 ns, single-shot qubit readout does not yield a meaningful fidelity. To enhance the signal-to-noise ratio, we average over many readout traces that have been down-modulated to 50 MHz, and then apply a fast Fourier transform to the averaged signal to extract its amplitude and phase.

B: Measurement Setup

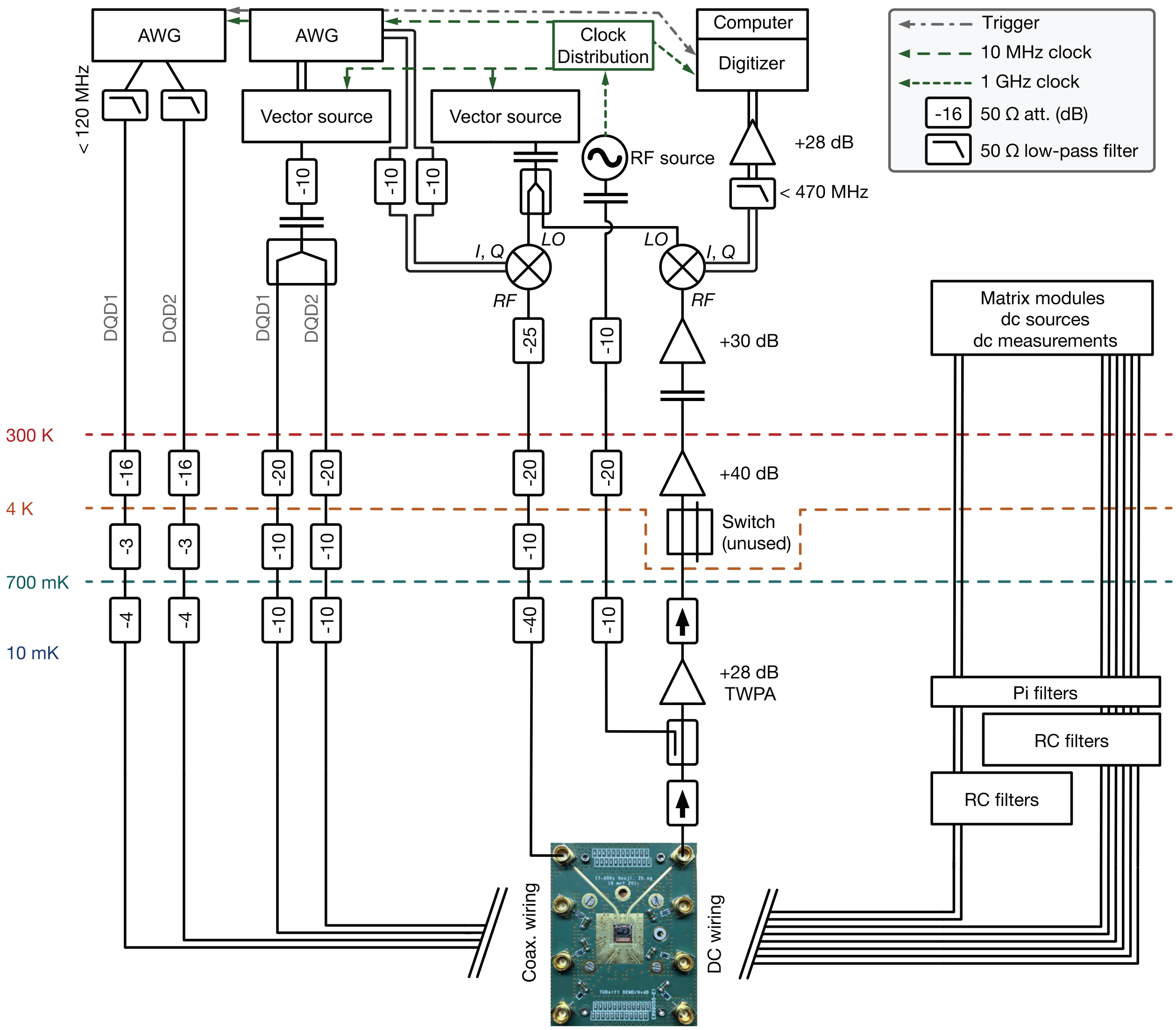

Fig. S2: Schematic overview of the measurement setup used in the experiment.

Resonator circuitry

An AWG (Tektronix AWG5014C) generates the I,Q signals for the resonator probe tones using a sample rate of 1 GS/s. The I,Q signals are attenuated by 10 dB to enhance the voltage resolution allowing for improved calibration of the I and Q amplitudes, compensating for possible asymmetries in the up-converting IQ mixer (Marki M1-0307LXP). The output of a vector source (Keysight E8267D) is splitted and provides the LO tone for this mixer as well as for the down-converting mixer. The RF output of the up-converting mixer then reaches the cryostat (Oxford Instruments Triton 400) and connects to the 'MW in' resonator feedline on the chip (Fig. 1) after various attenuation stages.

The returning signal from the 'MW out' feedline is then amplified by a TWPA, which has been configured for maximum gain (Fig. S3), after passing through an isolator (QuinStar 0xE89) and a directional coupler through which the TWPA pump tone enters. After passing another isolator, the signal is amplified at the 4K stage (Low Noise Factory LNF-LNC4-8A) and at room temperature (Miteq AFS3 10-ULN-R) after which it is down-converted to a signal of 50 MHz. The signal is then filtered by a <470 MHz low-pass filter before it is amplified once more (Stanford Research Systems SRS445A) and digitized (AlazarTech ATS9870) with a sample rate of 1 GS/s.

The RF source that generates the TWPA pump tone also supplies the reference clock signal to an in-house developed RF reference distribution unit. This unit shares the RF reference signal with the AWG's, vector sources and the digitizer in the setup to synchronize all clocks.

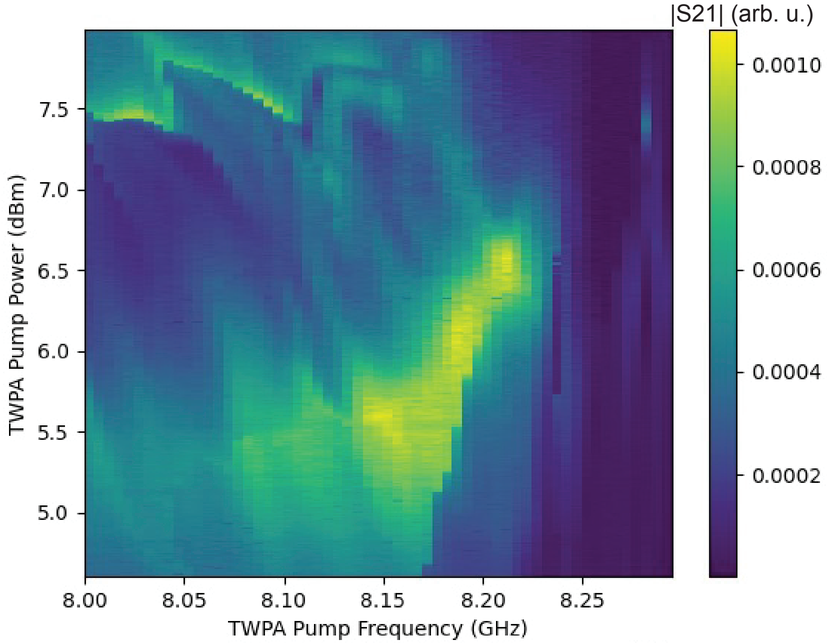

Fig. S3: TWPA calibration scan. In order to find the optimal operation point of the TWPA, a calibration scan is executed. The probe frequency is set to the resonator bare frequency of $\omega_r/2\pi = 6.9015$ GHz, with both electrons parked in single dots, thus decoupled from the resonator. Next, we sweep the RF frequency and RF power of the TWPA pump tone to map out the gain profile. Based on this measurement we choose the set point of the TWPA, which is a power of 5.17 dBm and a frequency of 8.086 GHz. These settings are used for every experiment reported in this work.

DQD-gates circuitry

The MW-bursts used to drive the qubits in the experiment are generated by using the internal IQ modulation of the vector source, where an AWG supplies the I,Q signals. The output of the vector source is splitted and connected to the LP_i gates (Fig. 1) after attenuation at the various stages. A separate AWG is used to generate the voltage pulses that allow us to quickly tune the DQD's into and out of the charge degeneracy point. The two lines from the AWG connect to the RP_i gates of both DQD's (Fig. 1) after attenuation at several stages. Home-built voltage sources used to DC-bias the DQD-gates are mounted in IVVI racks and are connected to various home-built matrix modules. The DC lines breaking out from the modules are filtered using home-built Pi filters and subsequently RC-filters before reaching the chip in the cryostat.

C: Parameter table

TABLE S1: Parameters used for the various measurements. "Measured" indicates a parameter is extracted directly from experiment. "Estimated" means the parameter is estimated based on modeling and on other parameters that are directly measured.

**General**	**Determination**	**Qubit 1**	**Qubit 2**	**Other**
$\omega_r/2\pi$	measured			6.9105 GHz
$g_c/2\pi$	estimated	192 MHz	192 MHz	
ΔB_x	estimated	42 mT	42 mT	
T_1	measured	200-260 ns	100-130 ns	
T_2^*	measured	60-80 ns	40-60 ns	
T_2^H	measured	140-160 ns	70-90 ns	
T_2^{Rabi}	measured	100-110 ns	100-110 ns	
**Fig. 2**				
$\chi_c/2\pi$	measured	13.5 MHz	13.5 MHz	
$g_s/2\pi$	estimated	21.5 MHz	21.5 MHz	
$\Delta_{2s}/2\pi$	measured	65.5 MHz	65.5 MHz	
t_c/h	estimated	4.8 GHz	4.8 GHz	
$\omega_q/2\pi$	measured	6.818 GHz	6.818 GHz	
**Fig. 3b,d**<br>**Fig. 4e-f** at $\phi = 11.2°$				
$\chi_c/2\pi$	measured	13.5 MHz	13.5 MHz	
$g_s/2\pi$	estimated	21.5 MHz	21.5 MHz	
$\Delta_{2s}/2\pi$	measured	65.5 MHz	65.5 MHz	
t_c/h	estimated	4.8 GHz	4.8 GHz	
$\omega_q/2\pi$	measured	6.818 GHz	6.818 GHz	
$2J/2\pi$	measured			init $\vert 10\rangle$: 11.6 ± 0.2 MHz<br>init $\vert 01\rangle$: 11.8 ± 0.2 MHz
**Fig. 4a-b**<br>**Fig. 5**				
$\chi_c/2\pi$	measured	20.5 MHz	20.5 MHz	
$g_s/2\pi$	estimated	31.9 MHz	31.9 MHz	
$\Delta_{2s}/2\pi$	measured	63 MHz	63 MHz	
t_c/h	estimated	4.35 GHz	4.35 GHz	
$\omega_q/2\pi$	measured	6.807 GHz	6.807 GHz	
$2J/2\pi$	measured			init $\vert 10\rangle$: 21.4 ± 0.3 MHz<br>init $\vert 01\rangle$: 21.3 ± 0.3 MHz
**Fig. 4c-d**				
$\chi_c/2\pi$	measured	20.5 MHz	20.5 MHz	
$g_s/2\pi$	estimated	31.9 MHz	31.9 MHz	
$\Delta_{2s}/2\pi$	measured	89 MHz	89 MHz	
t_c/h	estimated	4.35 GHz	4.35 GHz	
$\omega_q/2\pi$	measured	6.78 GHz	6.78 GHz	
$2J/2\pi$	measured			init $\vert 10\rangle$: 18.2 ± 0.4 MHz<br>init $\vert 01\rangle$: 18.7 ± 0.3 MHz

D: Simulations

The system presented in this study consists of two flopping-mode qubits, qubits encoded in individual electron spins confined inside a double quantum dot. Each electron spin is coupled to a single mode of a joint superconductor resonator. This composite system is well described by the following Hamiltonian

$$H = H_{\rm res} + H_{\rm DQD,1} + H_{\rm DQD,2} + H_{\rm int,1} + H_{\rm int,2}. \tag{S1}$$

The first term $H_{\rm res} = \hbar\omega_r a^\dagger a$ describes photons inside the resonator, where $a^\dagger$ (a) creates (annihilates) a photon with angular frequency ω_r. The second (third) term describes the dynamics of qubit 1 (2). Each flopping-mode qubit is modelled as a 4-level system [1, 2]

$$H_{\rm DQD,1(2)} = \frac{1}{2}\left[\epsilon_{1(2)}\tau_{z,1(2)} + 2t_{c,1(2)}\tau_{x,1(2)} + (\boldsymbol{h}_{1(2)} + \boldsymbol{\Delta h}_{1(2)}\tau_{z,1(2)}/2)\cdot\boldsymbol{\sigma}_{1(2)}\right] \tag{S2}$$

with $\boldsymbol{\sigma}_{1(2)} = (\sigma_{x,1(2)}, \sigma_{y,1(2)}, \sigma_{z,1(2)})^T$. Here, $\tau_{\xi,1(2)}$ and $\sigma_{\xi,1(2)}$ with $\xi = x, y, z$ are Pauli matrices describing the position and spin degree of freedom of the electron in DQD 1 (2). The parameter $\epsilon_{1(2)}$ denotes the energy detuning and $t_{c,1(2)}$ the tunnel coupling between the left and right quantum dot. The effect of the global and micromagnet-induced magnetic field in DQD 1 (2) is described by $\boldsymbol{h}_{1(2)} = \mu_B g_e(B_{L,1(2)} + B_{R,1(2)})/2$ and $\boldsymbol{\Delta h}_{1(2)} = \mu_B g_e(B_{L,1(2)} - B_{R,1(2)})$, with $g_e = 2$ being the Lande g-factor of an electron in silicon and μ_B Bohr's magneton, giving rise to a hybridization between spin and position (charge). A detailed characterisation of the parameters is given in Ref. [1].

The last terms in Hamiltonian (S1) describe the charge-photon interaction

$$H_{\rm int,1(2)} = \hbar g_{c,1(2)}\tau_{z,1(2)}(a^\dagger + a) \tag{S3}$$

with coupling strength $g_{c,1(2)}$. The spin-photon interaction is mediated via the charge degree of freedom.

Reduced models

Since running the full system is computationally expensive, we eliminate the charge degree of freedom in the limit $\max(\hbar g_{c1(2)}, |\boldsymbol{\Delta h}_{1(2)}|) \ll 2t_{c,1(2)}$ using standard block diagonalization methods [3–5]. The reduced Hamiltonian at $\epsilon_{1(2)} = 0$ then reads

$$H_{\rm res} \approx \hbar(\omega_r - \chi_1 - \chi_2)a^\dagger a, \tag{S4}$$

$$H_{\rm DQD,1(2)} \approx \frac{1}{2}\omega_{Q1(2)}\sigma_{z,1(2)}, \tag{S5}$$

$$H_{\rm int,1(2)} \approx \hbar g_{s,1(2)}(a^\dagger + a)\sigma_{x,1(2)}, \tag{S6}$$

where $\hbar\chi_{1(2)} = \hbar^2 g_{c,1(2)}^2\left(\frac{1}{2t_{c,1(2)} - \hbar\omega_r} + \frac{1}{2t_{c,1(2)} + \hbar\omega_r}\right)$ is the charge dispersive shift [6] and $g_{s,1(2)}$ is the spin-photon coupling. The resulting full system Hamiltonian is identical to the 2-qubit Dicke model, a quantum Rabi model with two qubits coupled to a common resonator mode. The Tavis-Cummings Hamiltonian in the main text follows from Hamiltonian Eq. (S6) under the rotating frame approximation $(a^\dagger + a)\sigma_{x,1(2)} \to (a^\dagger\sigma_{-,1(2)} + a\sigma_{+,1(2)})$ with $2\sigma_{\pm,1(2)} = \sigma_{x,1(2)} \pm i\sigma_{y,1(2)}$.

In the limit $g_{s,1(2)} \ll |\Delta_{Q1(2)}|$ with $\Delta_{Q1(2)} = \omega_r - \chi_1 - \chi_2 - \omega_{Q1(2)}$, the so-called dispersive regime, we can further eliminate the photonic degree of freedom. The final Hamiltonian then reads

$$H_{\rm disp} = \frac{\hbar}{2}\omega_{Q1}\sigma_{z,1} + \frac{\hbar}{2}\omega_{Q2}\sigma_{z,2} + \frac{\hbar J}{2}(\sigma_{x,1}\sigma_{x,2} + \sigma_{y,1}\sigma_{y,2}), \tag{S7}$$

with the interaction strength $2J = g_{s,1}g_{s,2}\left(\frac{1}{\Delta_{Q1}} + \frac{1}{\Delta_{Q2}}\right)$. Note that Eq. (S7) is identical to Eq. (1) of the main text using the identity $\sigma_{x,1}\sigma_{x,2} + \sigma_{y,1}\sigma_{y,2} = 2(\sigma_{+,1}\sigma_{-,2} + \sigma_{-,1}\sigma_{+,2})$.

Numerical simulations and noise models

The dynamics of the system can be computed by solving the Schrödinger equation

$$i\hbar\frac{d}{dt}\psi = H(t)\psi, \tag{S8}$$

where ψ is the quantum state. Additionally, each qubit is subject to relaxation and dephasing channels, the resonator is subject to photon decay, and the system parameters are subject to low-frequency fluctuations.

Qubit relaxation $\gamma_{r,Q1(2)}$, qubit dephasing $\gamma_{\phi,Q1(2)}$, and photon decay κ are introduced as Markovian processes within the Lindblad formalism by solving the resulting master equation

$$\frac{d}{dt}\rho = -\frac{i}{\hbar}(H\rho - \rho H) + \sum_i \mathcal{D}_i(\rho), \tag{S9}$$

where ρ is the density matrix, $\mathcal{D}_i(\rho) = \gamma_i(2L_i\rho L_i^\dagger - L_i^\dagger L_i\rho - \rho L_i^\dagger L_i)/2$ is the dissipation operator. Explicitly, we use the following channels

$$\gamma = \gamma_{r,Q1(2)} \quad L_{r,Q1(2)} = \sigma_{-,1(2)} \tag{S10}$$

$$\gamma = \gamma_{\phi,Q1(2)} \quad L_{\phi,Q1(2)} = \sigma_{z,1(2)} \tag{S11}$$

$$\gamma = \kappa \qquad L_\kappa = a\ . \tag{S12}$$

Readout model

We model the readout via the resonator within the input-output framework in the linear response regime [7]. The measured output signal is then given in the Hilbert-Schmidt or Liouville space by [8]

$$S_{21,Q1(2)} = i\hbar\sqrt{\kappa_{\rm in}\kappa_{\rm out}}\, \langle I_n|\,(a\otimes I_n)(-\mathcal{H}_{Q1(2)} - \mathcal{L} - i\hbar\omega_{\rm read} I_{n^2})^{-1}(a^\dagger \otimes I_n - I_n\otimes a^\star)\,|\rho_{\rm final}\rangle\,. \tag{S13}$$

Here, I_n is an identity matrix of dimension $n = \dim(H)$, $\mathcal{H}_{Q1(2)} = -i(H\otimes I_n - I_n \otimes H^\star)$ is the full Hamiltonian with $g_{s,2(1)} = 0$ and $\chi_{2(1)} = 0$, and $\mathcal{L} = \sum_i \gamma_i(2L_i\otimes L_i - I_n\otimes(L_i^T L_i)^T - (L_i^T L_i)\otimes I_n)/2$ is the Lindbladian of the system. The vector $|A\rangle$ is the vectorized form of the matrix A, $\rho_{\rm final}$ is the final density matrix, $\omega_{\rm read}$ is the readout frequency, and $\kappa_{\rm in(out)}$ is the coupling rate between the resonator and the input (output) line.

Fitting procedure

We use the following fitting procedures. We fit the qubit relaxation and dephasing times, $\gamma_{r,Q1(2)}$ and $\gamma_{\phi,Q1(2)}$, the spin-photon couplings of both DQD's, which we assume to be identical, and an amplitude and offset of the readout signal. All other inputs to the model are experimentally measured. Explicitly, these are the charge dispersive shifts χ_i, the resonator frequency and linewidth ω_r and κ_r, and the qubit frequencies ω_{Qi}. Extended Data Fig. 3 shows the fits of the full model to the exchange oscillations reported in the main text. The described measured respective input parameters for each panel can be seen in Table S1.

Note that here the g_s values fitted from Extended Data Fig. 3.a-b and e-f are very close to those estimated for Fig. 3 and Fig. 4 using the input-output theory whereas the g_s values fitted from Extended Data Fig. 3.c-d are slightly different. We can think of several possible contributions to such deviations. First, the magnetic field gradient is needed for the estimation based on input-output theory, but it can only be simulated and thus the number might be inaccurate. Second, in the fitted model, we have assumed that the qubits are perfectly at the same frequency. However, due to the slow electric drift in the device and consequentially the limited accuracy in the calibration, there could be a small difference in their frequencies which contributes to slightly different oscillation frequencies. Third, the slow drift can also impact the tunnel couplings and thus directly affect the g_s values. Finally, when the external magnetic field is changed, the magnetic field gradient from the micromagnet can also be somewhat different, which directly affects the spin-charge hybridization and thus the g_s.

Fidelity estimation

A calibrated time evolution under the iSWAP interaction Hamiltonian is expected to realize a two-qubit gate. Whereas the limited single-shot readout capabilities of the present device do not allow a direct measurement of the gate fidelity using for instance randomized benchmarking, we can estimate the fidelity of the two-qubit gate from the fit to the data based on the full model simulations. Explicitly, we compute the average gate fidelity of the two-qubit gate with respect to the iSWAP and $\sqrt{\text{iSWAP}}$ gate ignoring single-qubit phases

$$1 - F = \min_{\theta_{Q1},\theta_{Q2}} \frac{n^2 - \mathrm{tr}\left(\chi_{\rm sim}\left[U(\theta_{Q1},\theta_{Q2})\otimes U(\theta_{Q1},\theta_{Q2})^\dagger\right]\right)^\dagger}{n(n+1)}. \tag{S14}$$

Here, $n = 4$ and $U(\theta_{Q1}, \theta_{Q2}) = e^{-i\theta_{Q1}\sigma_{z,1}} e^{-i\theta_{Q2}\sigma_{z,2}} U_{\mathrm{iSWAP}(\sqrt{\mathrm{iSWAP}})}$ is the ideal target gate up to single-qubit phases with

$$U_{\mathrm{iSWAP}} = \begin{pmatrix} 1 & 0 & 0 & 0 \\ 0 & 0 & i & 0 \\ 0 & i & 0 & 0 \\ 0 & 0 & 0 & 1 \end{pmatrix} \tag{S15}$$

$$U_{\sqrt{\mathrm{iSWAP}}} = \begin{pmatrix} 1 & 0 & 0 & 0 \\ 0 & 1/\sqrt{2} & i/\sqrt{2} & 0 \\ 0 & i/\sqrt{2} & 1/\sqrt{2} & 0 \\ 0 & 0 & 0 & 1 \end{pmatrix}. \tag{S16}$$

Note that since the simulation is performed in the laboratory frame, the single-qubit phases simply account for the Larmor precession of each qubit.

The process matrix χ_{sim} can be extracted from the simulations using standard quantum process tomography techniques. Explicitly, we solve

$$|\rho_{\mathrm{red},ij}(t_g)\rangle\rangle = \chi_{\mathrm{sim}}(t)\,|\rho_{\mathrm{red},ij}(0)\rangle\rangle\,, \tag{S17}$$

where $\rho_{\mathrm{red},ij}(t) = \mathrm{tr}_{ph}(\rho_{ji}(t))$ is the ij-th vectorized reduced density matrix computed from the master equation by tracing out the photonic degree of freedom, for all input states $\rho_{ij} = |\psi_i\rangle\langle\psi_j|$ with $|\psi_i\rangle$ being the standard product basis $\{|00\rangle, |01\rangle\,|10\rangle, |11\rangle\}$.

E: Normalization

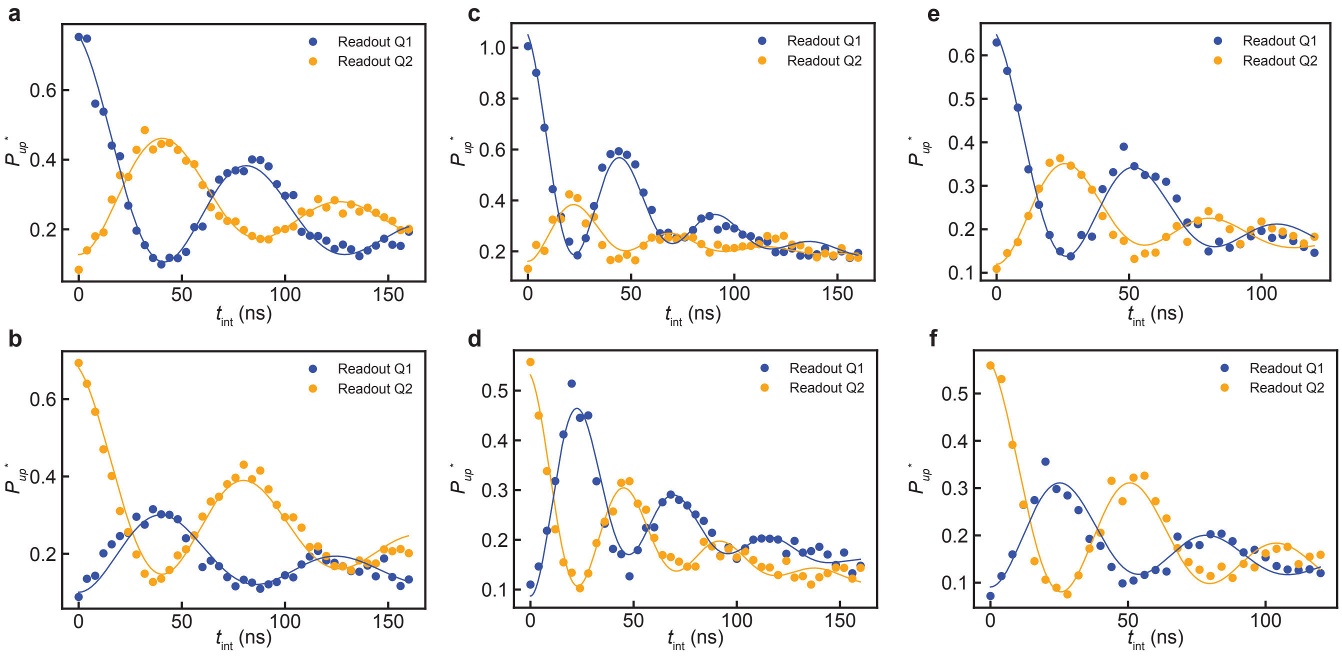

Fig. S4: iSWAP oscillations on a scale that has been normalized using Rabi oscillation data. Here, **a,b** correspond to Fig. 3b,d and **c-f** to Fig. 4a-d. These plots give an impression of P_{up} as function of interaction time. Due to the absence of single-shot readout in this device, single-qubit Rabi oscillations ($\Delta|S21|_{\mathrm{Rabi}}$) are used to normalize the iSWAP oscillation datasets ($\Delta|S21|_{\mathrm{exp}}$). The iSWAP oscillations are normalized using a Rabi oscillation taken with corresponding spin-photon coupling and spin-cavity detuning since the visibility of the Rabi oscillations may depend on the two-qubit operation regime. The normalization we used is expressed as $P^*_{up} = \frac{\Delta|S21|_{\mathrm{exp}} - \min(\Delta|S21|_{\mathrm{Rabi}})}{\max(\Delta|S21|_{\mathrm{Rabi}}) - \min(\Delta|S21|_{\mathrm{Rabi}})}$, where $\min(\Delta|S21|_{\mathrm{Rabi}})$ is the minimum value of the fitted Rabi oscillation, and $\max(\Delta|S21|_{\mathrm{Rabi}})$ the extrapolated maximum value of the Rabi oscillation. While the plots provide an indication of P_{up}, it is important to note the caveats associated with this normalization. First, the SPAM errors for the single-qubit Rabi and two-qubit interaction experiments can vary, and the resulting visibility differences lead to inaccurate normalization. These variations can occur due to slow electric drift of the device (see Supplementary Section D) introducing small spin frequency or spin-photon coupling differences between the two measurements, impacting state preparation. This mechanism could be behind P^*_{up} exceeding 1 in panel **c** (Readout Q1). Furthermore, the measurement visibility can vary due to the different delay between the drive and readout tones in both experiments, which can introduce different resonator photon population during the readout stages. Finally, the extrapolation of the Rabi data to zero burst time, used to estimate $\max(\Delta|S21|_{\mathrm{Rabi}})$, may not be accurate given that T_2^{Rabi} is not much longer than the Rabi period.

REFERENCES

[1] P. Harvey-Collard, J. Dijkema, G. Zheng, A. Sammak, G. Scappucci, and L. M. K. Vandersypen, Coherent spin-spin coupling mediated by virtual microwave photons, Physical Review X **12**, 021026 (2022).

[2] M. Benito, J. R. Petta, and G. Burkard, Optimized cavity-mediated dispersive two-qubit gates between spin qubits, Physical Review B **100**, 81412 (2019).

[3] M. Benito, X. Mi, J. M. Taylor, J. R. Petta, and G. Burkard, Input-output theory for spin-photon coupling in si double quantum dots, Physical Review B **96**, 235434 (2017).

[4] M. Benito, X. Croot, C. Adelsberger, S. Putz, X. Mi, J. R. Petta, and G. Burkard, Electric-field control and noise protection of the flopping-mode spin qubit, Physical Review B **100**, 125430 (2019).

[5] I. A. Day, S. Miles, H. K. Kerstens, D. Varjas, and A. R. Akhmerov, Pymablock: an algorithm and a package for quasi-degenerate perturbation theory (2024), arXiv:2404.03728 [quant-ph].

[6] S. Kohler, Dispersive readout: Universal theory beyond the rotating-wave approximation, Physical Review Applied **98**, 023849 (2018).

[7] J. Combes, J. Kerckhoff, and M. Sarovar, The SLH framework for modeling quantum input-output networks, Advances in Physics: X **2**, 784 (2017).

[8] T. Bonsen, P. Harvey-Collard, M. Russ, J. Dijkema, A. Sammak, G. Scappucci, and L. M. K. Vandersypen, Probing the Jaynes-Cummings Ladder with Spin Circuit Quantum Electrodynamics, Physical Review Letters **130**, 137001 (2023).
